# Direct Intra-Patient Comparison of Scaffold Protein-Based Tracers, [^99m^Tc]Tc-ADAPT6 and [^99m^Tc]Tc-(HE)_3_-G3, for Imaging of HER2-Positive Breast Cancer

**DOI:** 10.3390/cancers15123149

**Published:** 2023-06-11

**Authors:** Olga Bragina, Vladimir Chernov, Alexey Schulga, Elena Konovalova, Sophia Hober, Sergey Deyev, Jens Sörensen, Vladimir Tolmachev

**Affiliations:** 1 Department of Nuclear Therapy and Diagnostic, Cancer Research Institute, Tomsk National Research Medical Center, Russian Academy of Sciences, 634009 Tomsk, Russia; braginaod@onco.tnimc.ru (O.B.);; 2 Research Centrum for Oncotheranostics, Research School of Chemistry and Applied Biomedical Sciences, Tomsk Polytechnic University, 634050 Tomsk, Russia; schulga@gmail.com (A.S.); elena.ko.mail@gmail.com (E.K.); biomem@mail.ru (S.D.); 3 Shemyakin-Ovchinnikov Institute of Bioorganic Chemistry, Russian Academy of Sciences, 117997 Moscow, Russia; 4 Department of Protein Science, KTH Royal Institute of Technology, 100 44 Stockholm, Sweden; sophia@kth.se; 5 Department of Surgical Sciences, Nuclear Medicine & PET, Uppsala University, 751 85 Uppsala, Sweden; jens.h.sorensen@uu.se; 6 Department of Immunology, Genetics and Pathology, Uppsala University, 752 37 Uppsala, Sweden

**Keywords:** radionuclide molecular imaging, clinical study, HER2, scaffold proteins, DARPin, ADAPT6, technetium-99m

## Abstract

**Simple Summary:**

The receptor HER2 is overexpressed in some breast cancers. Tumours with a high HER2 expression can be successfully treated with the antibodies trastuzumab and pertuzumab. The radionuclide imaging of HER2 in disseminated cancer could help to select patients for treatment using these antibodies. Novel radiolabelled small-sized tracers, scaffold proteins, have shown excellent imaging properties in preclinical studies. The scaffold proteins [^99m^Tc]Tc-ADAPT6 and DARPin [^99m^Tc]Tc-(HE)_3_-G3 have been found to be safe in Phase I clinical trials. They showed promising results in the imaging of HER2. In this study, we compared the distribution of both tracers in the same patients with breast cancer to evaluate whether one of them has any decisive advantage. We found that both tracers provide an excellent visualization of tumours, but the accumulation of [^99m^Tc]Tc-ADAPT6 in tumours is higher. The data from this study are essential for researchers developing imaging agents.

**Abstract:**

Previous Phase I clinical evaluations of the radiolabelled scaffold proteins [^99m^Tc]Tc-ADAPT6 and DARPin [^99m^Tc]Tc-(HE)_3_-G3 in breast cancer patients have demonstrated their safety and indicated their capability to discriminate between HER2-positive and HER2-negative tumours. The objective of this study was to compare the imaging of HER2-positive tumours in the same patients using [^99m^Tc]Tc-ADAPT6 and [^99m^Tc]Tc-(HE)_3_-G3. Eleven treatment-naïve female patients (26–65 years) with HER2-positive primary and metastatic breast cancer were included in the study. Each patient was intravenously injected with [^99m^Tc]Tc-ADAPT6, followed by an [^99m^Tc]Tc-(HE)_3_-G3 injection 3–4 days later and chest SPECT/CT was performed. All primary tumours were clearly visualized using both tracers. The uptake of [^99m^Tc]Tc-ADAPT6 in primary tumours (SUVmax = 4.7 ± 2.1) was significantly higher (*p* < 0.005) than the uptake of [^99m^Tc]Tc-(HE)_3_-G3 (SUVmax = 3.5 ± 1.7). There was no significant difference in primary tumour-to-contralateral site values for [^99m^Tc]Tc-ADAPT6 (15.2 ± 7.4) and [^99m^Tc]Tc-(HE)_3_-G3 (19.6 ± 12.4). All known lymph node metastases were visualized using both tracers. The uptake of [^99m^Tc]Tc-ADAPT6 in all extrahepatic soft tissue lesions was significantly (*p* < 0.0004) higher than the uptake of [^99m^Tc]Tc-(HE)_3_-G3. In conclusion, [^99m^Tc]Tc-ADAPT6 and [^99m^Tc]Tc-(HE)_3_-G3 are suitable for the visualization of HER2-positive breast cancer. At the selected time points, [^99m^Tc]Tc-ADAPT6 has a significantly higher uptake in soft tissue lesions, which might be an advantage for the visualization of small metastases.

## 1. Introduction

An overexpression of the epidermal growth factor receptor type 2 (HER2) occurs in 15–20% of breast cancer patients and is associated with an unfavourable prognosis and aggressive course of the disease [1]. However, tumours with a high HER2 expression (clinically HER2-positive tumours) can be successfully treated using a targeted therapy with a monoclonal antibody trastuzumab, an antibody–drug conjugate trastuzumab–emtansine or a combination of the antibodies trastuzumab and pertuzumab [2,3]. The stratification of patients for such therapies mandates the determination of HER2 expression levels, preferably in all lesions. Currently, tumour biopsy samples are needed for the determination of HER2 status since immunohistochemistry (IHC) and fluorescence in situ hybridization (FISH) are the only methods recommended by the American Society of Clinical Oncology and College of American Pathologists (ASCO/CAP, 2018) [4]. Unfortunately, the invasiveness of biopsy does not permit the sampling of primary tumours and metastases in regional lymph nodes and distant organs simultaneously, which might be critical because of HER2 expression heterogeneity between the primary tumour and metastatic sites (up to 40% of all cases) [5,6]. 

To address the heterogeneity issue, molecular imaging using SPECT and PET has been actively studied for the evaluation of HER2 expression in breast cancer patients [7]. The most common approach for the development of HER2-imaging probes is the use of radiolabelled therapeutic monoclonal antibodies and their derivatives [8]. The major issue with the use of antibodies as imaging probes is their slow blood clearance, which permits a reasonable imaging contrast only 4–5 days after injection. As an alternative to monoclonal antibodies, engineered scaffold proteins were proposed for radionuclide imaging [9,10,11]. Because of their small size, unbound scaffold proteins are cleared rapidly from the blood and healthy tissues, enabling imaging at the day of injection. The scaffold proteins designed ankyrin repeat proteins (DARPins) and albumin-binding domain-derived affinity proteins (ADAPTs) are characterized by their small size, high stability, and high affinity and specificity for selected molecular targets. DARPin G3 is based on the ankyrin scaffold and is characterized by its low molecular weight of 14 kDa and high affinity for the epidermal growth factor receptor 2 type HER2 (90 pM) [12,13,14]. ADAPTs were developed using a 46-amino acid framework derived from an albumin-binding domain (ABD), which spontaneously folds into a three-helical structure that does not depend on disulfide bridges. ADAPT6 has a small size (molecular weight 5–7 kDa) and a high affinity for the HER2 receptor (1 nM) [15,16,17]. 

Completed Phase I clinical trials of [^99m^Tc]Tc-ADAPT6 (ClinicalTrials.gov ID: NCT03991260, https://clinicaltrials.gov/ct2/show/NCT03991260 (accessed on 8 June 2023)) and [^99m^Tc]Tc-(HE)_3_-G3 (ClinicalTrials.gov ID: NCT04277338, https://clinicaltrials.gov/ct2/show/NCT04277338 (accessed on 8 June 2023)) in breast cancer patients with both positive and negative expression of HER2 have demonstrated that the injections of both proteins are well-tolerated, not associated with any adverse effects and result in a low radiation dose burden in patients. In addition, both [^99m^Tc]Tc-ADAPT6 and [^99m^Tc]Tc-(HE)_3_-G3 have significantly (*p* < 0.05, Mann–Whitney test) higher tumour-to-contralateral site ratios for clinically HER2-positive primary breast tumours compared to HER2-negative, which indicates that they could be used for the stratification of patients for HER2-targeting therapies. The optimal imaging time was 2 h after injection for [^99m^Tc]Tc-ADAPT6 and 4 h after injection for [^99m^Tc]Tc-(HE)_3_-G3 [9,10]. It has to be noted that these studies were performed using stand-alone SPECT scanners, which complicated the quantitative evaluation of the tracers’ uptake in tumours. Contemporary SPECT/CT cameras permit reasonably good attenuation corrections and enable the calculation of standard uptake values (SUV) with acceptable accuracy. A comparative quantitative evaluation of the tumour uptake of ADAPTs and DARPins should provide a better understanding of the advantages and disadvantages of their use in clinics.

According to the clinical trial registration, “The primary aim of this study was to compare SPECT/CT imaging properties of [^99m^Tc]Tc-ADAPT6 and [^99m^Tc]Tc-(HE)_3_-G3 in HER2-positive primary breast tumours of the same patients before systemic (chemo/targeted therapy) treatment. The second aim was to compare the SPECT/CT tumour imaging data with the data concerning HER2 expression obtained by immunohistochemistry (IHC) and/or fluorescent in situ hybridization (FISH) analysis of biopsy samples.” 

## 2. Materials and Methods

### 2.1. Patients

This was a prospective, open-label and non-randomized diagnostic study of eleven female patients (26–65 years) with HER2-positive primary and metastatic breast cancer before systemic (chemotherapy and targeted therapy) treatment (ClinicalTrials.gov Identifier: NCT05376644). The study was performed in line with the principles of the Declaration of Helsinki. Approval was granted by the Scientific Council of Cancer Research Institute and Board of Medical Ethics, Tomsk National Research Medical Center of the Russian Academy of Sciences (№ 6, 4 March 2022. Written informed consent was obtained from the patients. The authors affirm that human research participants provided informed consent for the publication of images.

According to the clinical trial registration, “Inclusion criteria were: over 18 years of age; diagnosis of primary breast cancer with possible lymph node metastases; availability of results from HER2 status test previously determined using biopsy material from a primary tumour: HER2-positive, defined as a DAKO HercepTest™ score of 3+ or FISH positive; haematological, liver and renal function test results within the following limits: white blood cell count: >2.0 × 10^9^/L, haemoglobin: >80 g/L, platelets: >50.0 × 10^9^/L, ALT, ALP, AST: ≤5.0 times Upper Limit of Normal; bilirubin ≤ 2.0 times Upper Limit of Normal; serum creatinine: Within Normal Limits. A negative pregnancy test was requested from all patients of childbearing potential. Sexually active women of childbearing potential participating in the study had to use a medically acceptable form of contraception for at least 30 days after study termination. Subjects had to be capable to undergo the diagnostic investigations planned in the study.

Exclusion criteria were: a second, non-breast malignancy; active current autoimmune disease or history of autoimmune disease; active infection or history of severe infection within the previous 3 months (if clinically relevant at screening); known positive HIV test or chronically active hepatitis B or C; administration of other investigational medicinal product within 30 days of the screening; ongoing toxicity > grade 2 from previous standard or investigational therapies, according to US National Cancer Institute criteria.” 

**Figure 1:**
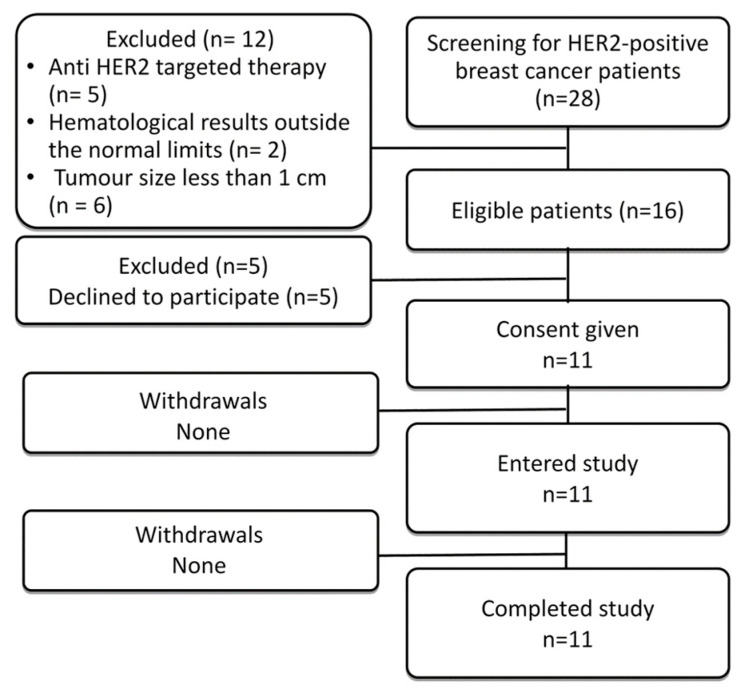
Flow diagram according to Standards for Reporting of Diagnostic Accuracy Studies (STARD).

In total, eleven patients were enrolled into the study (Figure 1; Table 1). Before imaging using radiolabelled scaffold proteins, a mammogram (Giotto Image), bone scan (Siemens Symbia Intevo Bold) with ^99m^Tc-pyrophosphate, chest CT (Siemens Somatom Emotions 16 ECO) and ultrasound imaging of the breast, regional lymph nodes and liver (GE LOGIQ E9) were performed for all patients. Abdomen CT was additionally performed for patients 1 and 5.

### 2.2. HER2 Immunohistochemistry

The HER2 status in primary tumours was evaluated using core biopsy material according to the guidelines of the American Society of Clinical Oncology and College of American Pathologists (ASCO/CAP, 2018). IHC for HER2 detection was performed with a HercepTest using a DAKO autostainer according to the manufacturer’s instructions. Fluorescent in situ hybridization was performed using LSI HER2 (17q12)/CEP17 probe (Leica) for patient 6. The tumours were classified as HER2-positive in the case of an IHC score 3+ or IHC score 2+ and FISH-positive test. Lymph node (LN) metastases were confirmed by histology using core biopsy in all patients. The sizes of the primary tumour and metastatic lymph nodes were measured using an ultrasound.

### 2.3. Radiochemistry

The labelling of [^99m^Tc]Tc-ADAPT6 and [^99m^Tc]Tc-(HE)_3_-G3 was performed in aseptic conditions, immediately before injection, according to the methods described earlier [14,17]. The CRS (Center for Radiopharmaceutical Sciences) kit was used for converting ^99m^Tc-pertechnetate to [^99m^Tc]Tc(H_2_O)_3_(CO)_3_^+^. Labelled proteins were purified via size-exclusion chromatography. An analysis was performed using instant thin layer chromatography (Agilent Technologies, Santa Clara, CA, USA). The mobile phases were PBS (Rf = 0 for the radiolabelled proteins and [^99m^Tc]TcO_2_.nH_2_O; Rf = 1 for [^99m^Tc]Tc(H_2_O)_3_(CO)_3_^+^ and [^99m^Tc]TcO^4−^)) and pyridine–acetic acid–water, 10:6:3 (Rf = 0 for [^99m^Tc]TcO_2_.nH_2_O and Rf = 1 for the radiolabelled proteins, [^99m^Tc]Tc(H_2_O)_3_(CO)_3_^+^ and [^99m^Tc]TcO^4−^). The radiochemical yield and purity for [^99m^Tc]Tc-ADAPT6 were 75 ± 24 and 97 ± 1%, respectively. The radiochemical yield and purity for [^99m^Tc]Tc-(HE)_3_-G3 were 88 ± 18 and 97 ± 2%, respectively.

### 2.4. SPECT/CT Protocol

Each patient was injected with [^99m^Tc]Tc-ADAPT6, followed by injection with [^99m^Tc]Tc-(HE)_3_-G3 after 3–4 days. In patient 1, the interval between injection was 15 days.

The injected protein masses and timing of imaging were selected based on the results of previous Phase I studies [9,10]. The injected protein masses were 500 and 3000 µg for [^99m^Tc]Tc-ADAPT6 and [^99m^Tc]Tc-(HE)_3_-G3, respectively. [^99m^Tc]Tc-ADAPT6 (523 ± 211 MBq) and [^99m^Tc]Tc-(HE)_3_-G3 (443 ± 185 MBq) were injected intravenously. Imaging was performed using a Siemens Symbia Intevo Bold scanner equipped with a high-resolution low-energy collimator. SPECT/CT scans (SPECT: 60 projections, 20 s each, stored in 256 × 256 pixel matrix/CT: 130 kV, effective 36 mAs) were performed 2 h after injection for [^99m^Tc]Tc-ADAPT6 and 4 h after injection for [^99m^Tc]Tc-(HE)_3_-G3 in all patients. SPECT images were reconstructed using a reconstruction xSPECT (Siemens) protocol based on the ordered subset conjugate gradient (OSCG) method (24 iterations, 2 subsets). The 3D Gaussian FWHM 10 mm filter (Soft Tissue) was used. The images were processed using the proprietary software package Syngo.via (Siemens).

### 2.5. SPECT Image Quantification

Maximal standard uptake values (SUVmax) were calculated in primary tumours, lymph node and liver metastases. SUVmax was also detected in contralateral symmetric regions to determine the tumour-to-contralateral site ratio (as a measure of a contrast) and in metastasis-free areas in the liver to calculate the liver metastases-to-liver ratio. For the evaluation of uptake in normal organs, which can contribute to the background in typical metastatic sites, the uptake (SUVmax) was also measured in the non-involved lymph nodes, lungs (segment 3 of the right lung in the projection of the aortic arch) and bones (fifth thoracic vertebra).

### 2.6. Statistics

The values are reported as mean and standard deviation. To compare values for the uptake and derived parameters, a paired *t*-test was used. A *p*-value less than 0.05 was considered statistically significant.

## 3. Results

**Figure 2:**
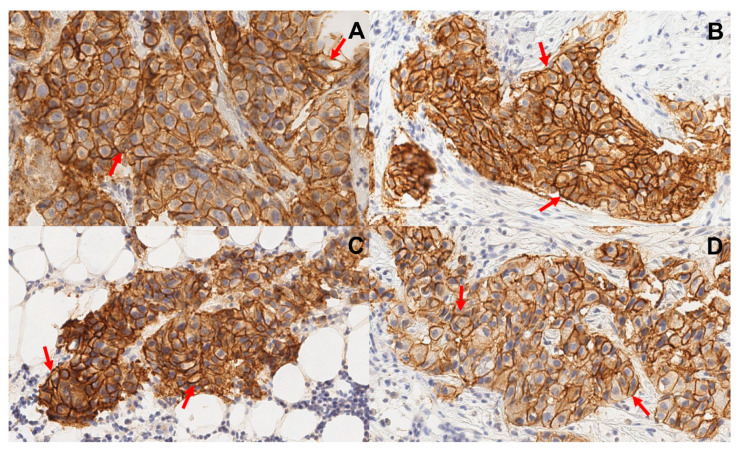
Representative images of immunohistochemistry staining of formalin-fixed and paraffin-embedded biopsy samples from primary tumours (**A**,**B**) and lymph node metastases (**C**,**D**). Samples from patients 5 (**A**,**C**) and 8 (**B**,**D**). Sections were stained using HercepTest. Multiplication factor ×20. Samples show uniform membrane staining of neoplastic cells. Arrows point at typical cells.

Eleven patients with HER-positive primary breast cancer were enrolled in the study (Figure 1; Table 1). The HER2 expression in all primary tumours was 3+ according to IHC. In addition, eight patients had HER2-positive lymph node metastases, in which HER2 status was verified by a core biopsy and subsequent histology analysis. Representative examples of IHC are provided in Figure 2. Two patients (1 and 5) had hepatic metastases., At enrolment, patient 1 had disseminated disease with multiple distant metastases.

**Figure 3:**
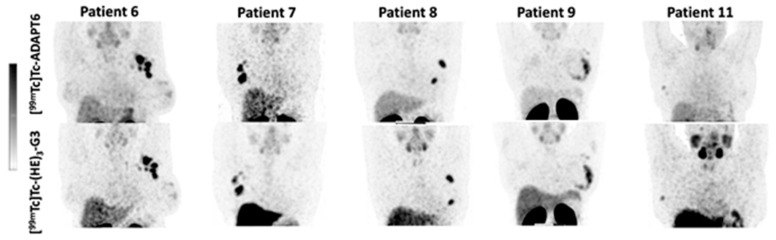
Comparison of SPECT images (maximum intensity projections) obtained using [^99m^Tc]Tc-ADAPT6 (2 h after injection) and [^99m^Tc]Tc-(HE)_3_-G3 (4 h after injection) in patients with HER2-positive breast cancer. The upper setting of a linear intensity scale is adjusted to SUV 6.8 in all images.

**Figure 4:**
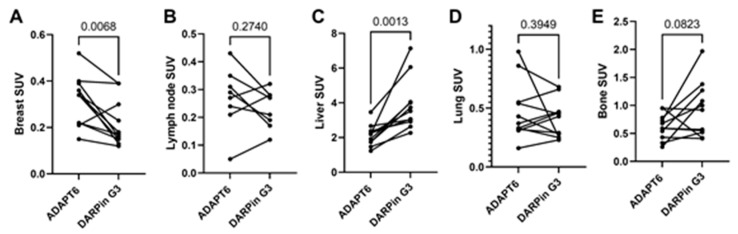
Uptake (SUV) in normal tissues after injection of [^99m^Tc]Tc-ADAPT6 (2 h) and [^99m^Tc]Tc-(HE)_3_-G3 (h). (**A**). Breast. (**B**). Non-involved lymph node. (**C**). Liver. (**D**). Lung (segment 3 of the right lung in the projection of the aortic arch). (**E**). Bone (5 thoracic vertebra).

The distribution of [^99m^Tc]Tc-ADAPT6 and [^99m^Tc]Tc-(HE)_3_-G3 in the scans is presented in Figure 3 and Figure 4. In normal tissues, the highest uptake was found in the kidneys (when in field of view) for both imaging agents. Another organ with high uptake was the liver, where the uptake of [^99m^Tc]Tc-(HE)_3_-G3 was visibly higher than the uptake of [^99m^Tc]Tc-ADAPT6 (Figure 3). An analysis of paired SUVmax measurements (Figure 4) confirmed that the hepatic uptake of [^99m^Tc]Tc-(HE)_3_-G3 (4.1 ± 1.6) was significantly (*p* < 0.005, paired *t*-test) higher than the uptake of [^99m^Tc]Tc-ADAPT6 (2.1 ± 0.7) at the selected time points. Further, the uptake of [^99m^Tc]Tc-ADAPT6 in the non-involved breast (SUVmax 0.3 ± 0.1) was significantly (*p* < 0.01, paired *t*-test) higher than the uptake of [^99m^Tc]Tc-(HE)_3_-G3 (SUVmax 0.2 ± 0.1). There was no significant difference between the uptake of tracers in non-involved lymph nodes (*p* > 0.05, paired *t*-test). The uptake in normal bones and lungs was also measured because these organs are typical metastatic sites for breast cancer. In these tissues, the accumulation of the tracer might influence the contrast of the imaging of bone and lung metastases. The uptake of the two tracers in normal lungs (SUVmax 0.4 ± 0.2 and 0.4 ± 0.1 for [^99m^Tc]Tc-ADAPT6 and [^99m^Tc]Tc-(HE)_3_-G3, respectively) and bones (SUVmax 0.6 ± 0.2 and 0.9 ± 0.5 for [^99m^Tc]Tc-ADAPT6 and [^99m^Tc]Tc-(HE)_3_-G3, respectively) did not differ significantly (*p* > 0.05, paired *t*-test). The uptake of the activity was also visible in the salivary glands and thyroid for both tracers.

**Figure 5:**
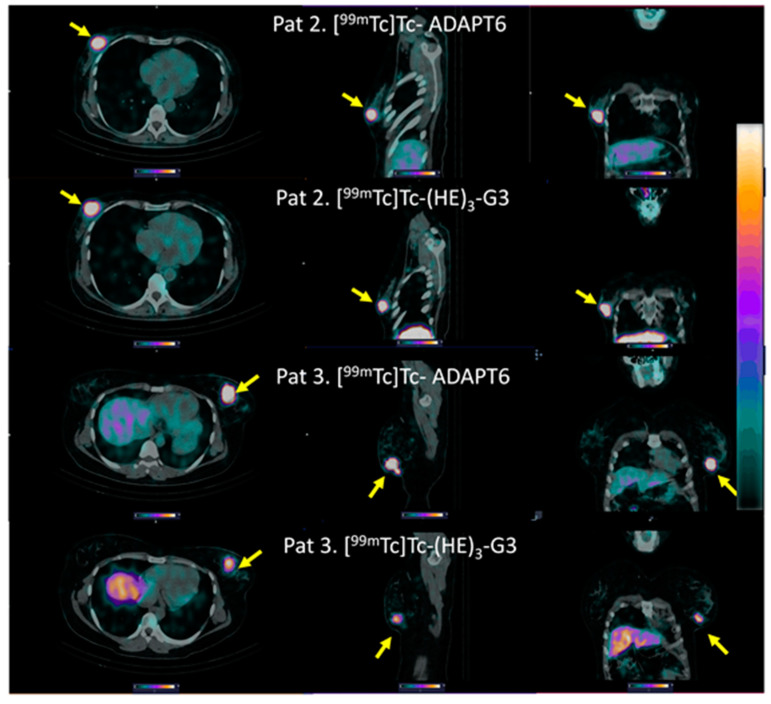
Representative SPECT/CT images of primary HER2-positive tumours after injection of [99mTc]Tc-ADAPT6 (2 h after injection) and [99mTc]Tc-(HE)_3_-G3 (4 h after injection). Arrows point at tumours. The upper setting of a linear intensity scale is adjusted to SUV 6.8 in all images.

**Figure 6:**
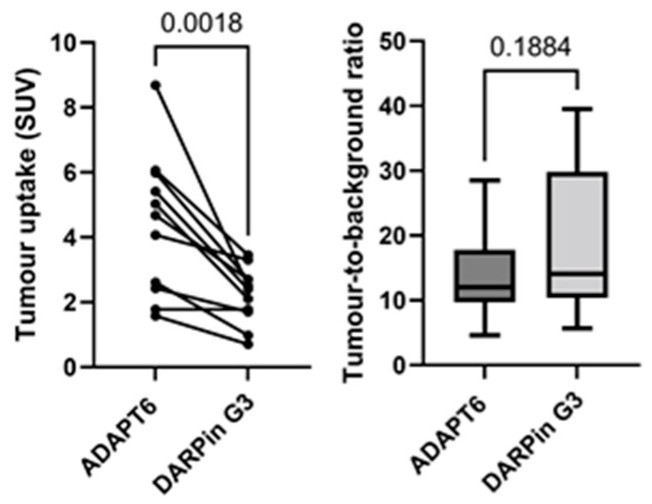
Uptake (SUV) in primary HER2-positive tumours and tumour-to-contralateral site ratio in HER2-positive breast cancer after injection of [^99m^Tc]Tc-ADAPT6 (2 h after injection) and [^99m^Tc]Tc-(HE)_3_-G3 (4 h after injection).

Both [^99m^Tc]Tc-ADAPT6 and [^99m^Tc]Tc-(HE)_3_-G3 enabled the clear visualization of all primary HER2-expressing tumours (representative images are presented in Figure 5). The mean tumour uptake of [^99m^Tc]Tc-ADAPT6 (SUVmax = 4.7 ± 2.1) was significantly (*p* < 0.005, paired *t*-test) higher than the mean tumour uptake of [^99m^Tc]Tc-(HE)_3_-G3 (SUVmax = 3.5 ± 1.7) (Figure 6). However, the tumour-to-contralateral site ratios (15.2 ± 7.4 for [^99m^Tc]Tc-ADAPT6 and 19.6 ± 12.4 for [^99m^Tc]Tc-(HE)_3_-G3) did not differ significantly (*p* > 0.05, paired *t*-test) (Figure 6).

**Figure 7:**
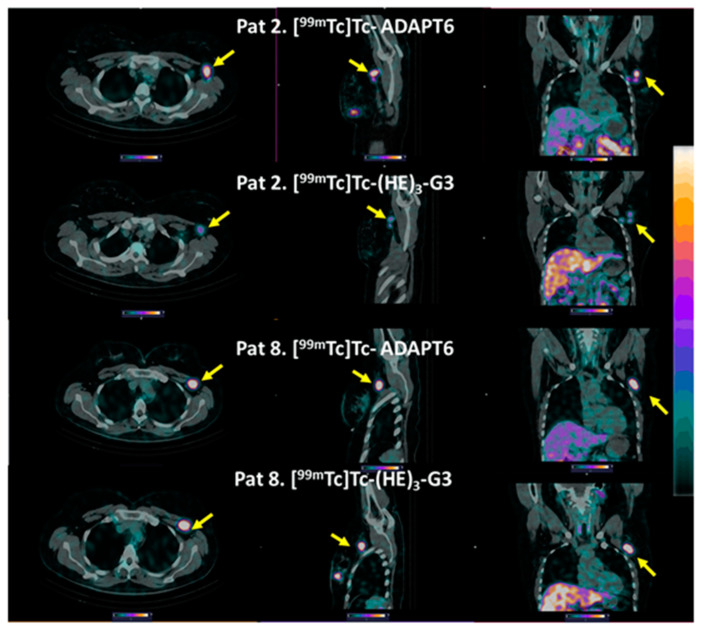
Representative SPECT/CT images of HER2-positive lymph node metastases after injection of [^99m^Tc]Tc-ADAPT6 (2 h after injection) and [^99m^Tc]Tc-(HE)_3_-G3 (4 h after injection). Arrows point at metastases. The upper setting of a linear intensity scale is adjusted to SUV 6.8 in all images.

All known lymph node metastases were visualized using both [^99m^Tc]Tc-ADAPT6 (2 h after injection) and [^99m^Tc]Tc-(HE)_3_-G3 (4 h after injection) (see representative images in Figure 7).

**Figure 8:**
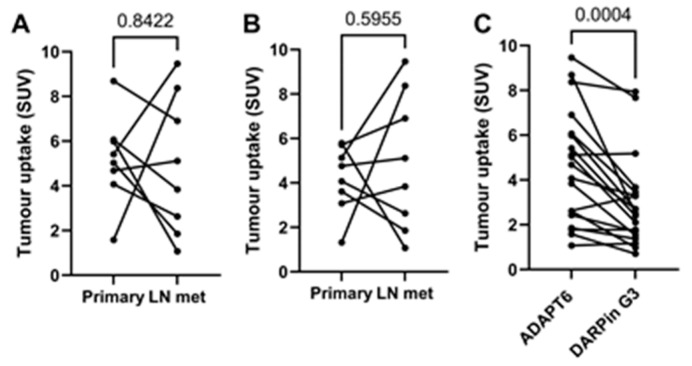
Relationship between uptakes in primary tumours and lymph node metastases for [^99m^Tc]Tc-ADAPT6 (**A**) and [^99m^Tc]Tc-(HE)_3_-G3 (**B**). Relationship between uptakes in all extrahepatic soft tissue lesions for [_99m_Tc]Tc-ADAPT6 and [^99m^Tc]Tc-(HE)_3_-G3 (**C**).

There was a noticeable difference between uptake values in some primary tumours and the corresponding lymph nodes (Figure 8A,B), but no correlation between the size of paired lesions and uptake value was found, which permitted the exclusion of the partial volume effect. Overall, the uptake of [^99m^Tc]Tc-ADAPT6 in all extrahepatic lesions was significantly (*p* < 0.0004) higher than the uptake of [^99m^Tc]Tc-(HE)_3_-G3 (Figure 8C).

**Figure 9:**
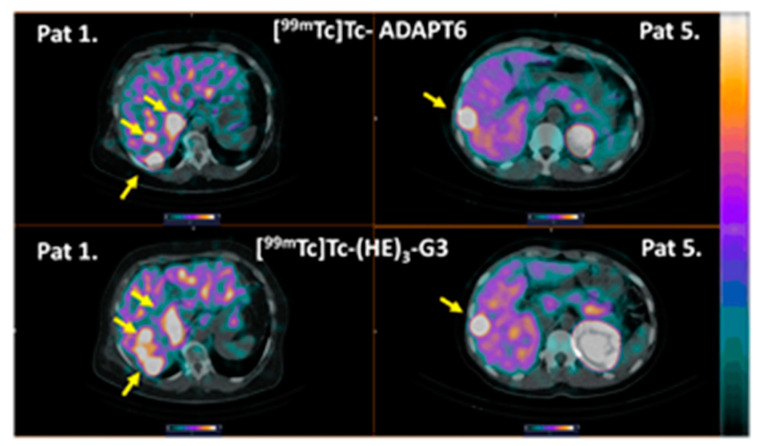
Imaging of liver metastases in HER2-positive breast cancer patients 1 and 5. Arrows point at metastases. The upper setting of a linear intensity scale is adjusted to SUV 6.8 in all images.

**Figure 10:**
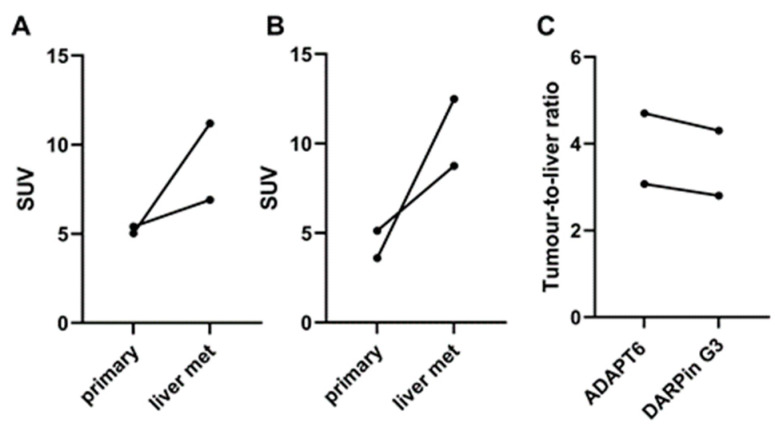
Comparison of accumulation of [^99m^Tc]Tc-ADAPT6 (**A**) and [^99m^Tc]Tc-(HE)_3_-G3 (**B**) in primary tumours and liver metastases (liver mets). (**C**) Liver metastases-to-liver ratio in HER2-positive breast cancer patients.

Liver metastases were detected in patients 1 and 5 during the examination (Figure 9). The foci of the elevated tracers’ accumulation were in agreement with the positions of the hepatic metastases in the CT images. Due to the refusal of patients to undergo a core biopsy, there was no morphological verification of liver metastases in any of the cases. In both patients, the uptake of [^99m^Tc]Tc-ADAPT6 and [^99m^Tc]Tc-(HE)_3_-G3 in liver metastases was higher than in primary tumours (Figure 10). In patient 1, the uptake in the liver metastasis was 1.3- and 1.7-fold higher than in the primary tumour, for [^99m^Tc]Tc-ADAPT6 and [^99m^Tc]Tc-(HE)_3_-G3, respectively. In patient 5, the uptake in the liver metastasis was 2.2- and 3.5-fold higher than in primary tumour, for [^99m^Tc]Tc-ADAPT6 and [^99m^Tc]Tc-(HE)_3_-G3, respectively. In both cases, [^99m^Tc]Tc-ADAPT6 provided approximately 10% higher liver metastases-to-liver ratio than [^99m^Tc]Tc-(HE)_3_-G3 (Figure 10C). 

**Figure 11:**
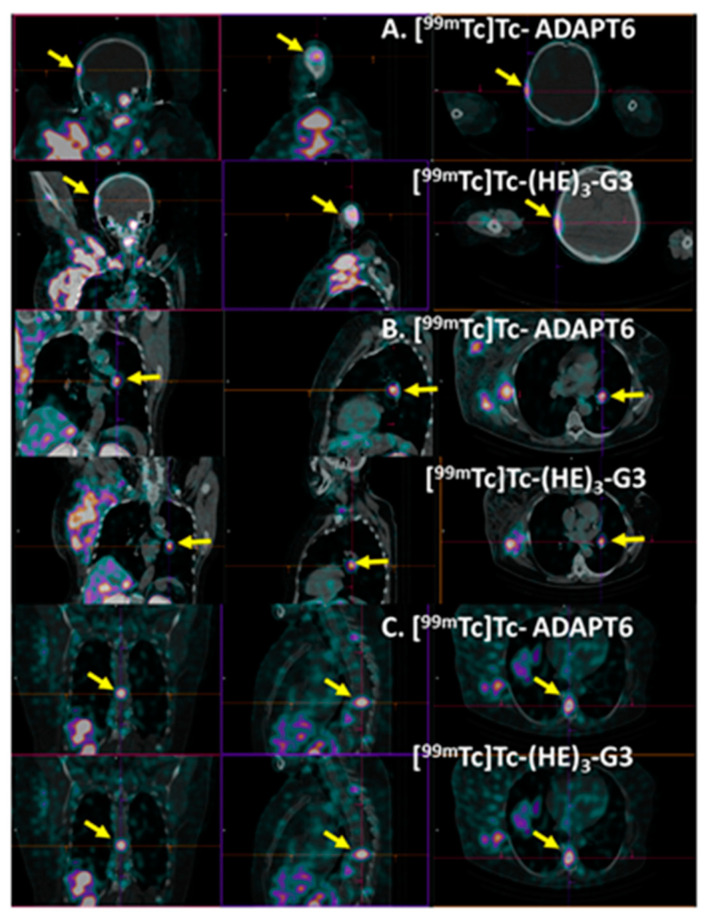
Visualization of metastases in (**A**) right parietal bone, (**B**) bronchopulmonary lymph node and (**C**) thoracic vertebra Th9 of patient 1 using [^99m^Tc]Tc-ADAPT6 (**A**) and [^99m^Tc]Tc-(HE)_3_-G3. Other metastases and very large primary tumours might also be in the field of view. Arrows point at metastases.

Patient 1 had inflammatory breast cancer and multiple distant metastases. Figure 11 shows examples of the metastases, which were visualized in the right parietal bone (SUVmax was 2.39 and 2.01 for [^99m^Tc]Tc-ADAPT6 and [^99m^Tc]Tc-(HE)_3_-G3, respectively), bronchopulmonary lymph node (SUVmax was 4.63 and 4.9 for [^99m^Tc]Tc-ADAPT6 and [^99m^Tc]Tc-(HE)_3_-G3, respectively) and thoracic vertebra (SUVmax was 4.38 and 5.06 for [^99m^Tc]Tc-ADAPT6 and [^99m^Tc]Tc-(HE)_3_-G3, respectively). The presence of these lesions was confirmed by CT. Morphologic verification was not performed because it was clinically unnecessary.

## 4. Discussion

Radiolabelled engineered scaffold proteins offer an advantage in the quantitative in vivo visualization of the expression of therapeutic molecular targets at the day of injection, which triggers an interest in this class of imaging agents [18,19]. While the common features of the scaffold proteins are small (compared to antibodies) size and high affinity, their structures are very different. One might expect that this could affect their uptake in normal tissues and in tumours. Understanding the differences in biodistribution between different scaffold proteins should facilitate the selection of the most appropriate molecules for further clinical development. A direct preclinical in vivo comparison of [^99m^Tc]Tc-ADAPT6 and [^99m^Tc]Tc-(HE)_3-_G3 revealed a significantly higher uptake of both radiopharmaceuticals in HER2-positive compared to HER2-negative tumours [20]. However, [^99m^Tc]Tc-ADAPT6 provided better discrimination between HER2-positive and HER2-negative xenografts. On the other hand, [^99m^Tc]Tc-(HE)_3-_G3 was capable of sensing the decrease in HER2 expression in response to trastuzumab therapy, which would be suitable for monitoring the early response to such treatment [20]. Still, there are numerous differences in the physiology and biochemistry of animal models and humans, and animal studies do not reflect all the interactions of scaffold proteins with targets that are expressed in normal tissues or their off-target interactions. Phase I clinical investigations of [^99m^Tc]Tc-ADAPT6 and [^99m^Tc]Tc-(HE)_3-_G3 in breast cancer patients showed that both radiopharmaceuticals were well-tolerated in humans and discriminated HER2-positive and HER2-negative tumours [9,10]. This opened a possibility to compare these two scaffolds in patients. 

This study was designed for a direct intrapatient comparison of the distribution and targeting properties of [^99m^Tc]Tc-ADAPT6 and [^99m^Tc]Tc-(HE)_3-_G3 in HER2-positive breast cancer patients before systemic treatment with trastuzumab in combination with chemotherapy. Imaging using both tracers was performed within 3 to 4 days (except in patient 1), minimizing the possibility of a HER2-expression change in tumours between scans. Thus, the study was informative even with a limited number of patients. Progress in the development of SPECT/CT scanners resulted in a radical improvement in radioactivity concentration quantification using single-photon imaging [21]. Phantom studies have shown that the Siemens Symbia Intevo camera, which was used in the current study, enables absolute activity and activity concentration measurements with high accuracy [22]. Overall, the study design should provide an accurate semiquantitative comparison between [^99m^Tc]Tc-ADAPT6 and [^99m^Tc]Tc-(HE)_3-_G3. A major issue in the study design was a noticeable dose burden due to performing CT scans for attenuation correction. Therefore, we had to limit evaluation to a single time point. For this reason, we selected the time point showing the best discrimination between HER2-positive and HER2-negative tumours in previous studies, i.e., 2 h after injection for [^99m^Tc]Tc-ADAPT6 and [^99m^Tc]Tc-(HE)_3-_G3 [9,10]. The dosing of the tracer was also selected based on the Phase I studies’ data. 

The sensitivity of molecular radionuclide imaging depends, to a high extent, on the imaging contrast, i.e., on the ratio of accumulation in tumours/metastases and surrounding tissues. Thus, low uptakes in normal organs are important. Particularly essential is the low uptake in lymph nodes, which are often involved in breast cancer, as well as in the bone, liver and lung, which are preferable sites for metastases in HER2-positive breast cancer [23]. At the selected time points, the difference between the tracers’ uptake in non-involved lymph nodes (Figure 4B) and lungs (Figure 4D) was not significant. Despite the tendency of a lower uptake of [^99m^Tc]Tc-ADAPT6 in bones, the difference in the uptake of [^99m^Tc]Tc-(HE)_3-_G3 was also not significant (*p* = 0.082, paired *t*-test). The hepatic uptake was significantly (*p* < 0.005, paired *t*-test) higher for [^99m^Tc]Tc-(HE)_3-_G3 (Figure 3 and Figure 4D). These data are in agreement with previous studies, reporting a hepatic uptake of 3.2 ± 1.1 and 5 ± 2 %ID per organ, 2 h after injection for [^99m^Tc]Tc-ADAPT6 (2 h) and 4 h for [^99m^Tc]Tc-(HE)_3-_G3 (4 h), respectively [9,10]. Thus, the distribution of [^99m^Tc]Tc-ADAPT6 is somewhat more favourable. It has to be noted that the uptake of another promising agent for the visualization of HER2, a single domain antibody (sdAb) [^99m^Tc]Tc-NM-02 ([^99m^Tc]Tc-RAD201) has shown a hepatic uptake of more than 10 %ID during the first day after injection [24,25]. The liver uptake for another scaffold protein, ^68^Ga-labelled affibody molecule ABY-025, was approximately SUV 5, when 500 µg was injected [26]. 

An interesting feature of both tracers was a noticeable uptake in salivary glands (Figure 3). This might be considered as a sign of the presence of radiopertechnetate due to either non-complete purification or release during catabolism, as salivary glands express a Na/I-symporter capable of taking up the pertechnetate anion. However, a prominent uptake of activity is observed not only for ^99m^Tc- or ^131^I-labeleled small imaging probes, such as [^99m^Tc]Tc-NM-02 ([^99m^Tc]Tc-RAD201) sdAb [24,25] or [^131^I]I-GMIB-Anti-HER2-VHH1 [27], but also for tracers labelled with other nuclides, such as ^111^In- and ^68^Ga-labelled affibody ABY-025 [28,29], or ^68^Ga- and ^18^F-labeled nanobodies [30,31]. Free radionuclides or radiocatabolites should not accumulate in salivary glands in the case of such labels. Most likely, the accumulation is caused by interaction of [^99m^Tc]Tc-ADAPT6 and [^99m^Tc]Tc-(HE)_3_-G3 with HER2, which is expressed in salivary glands. 

Both tracers visualized all known primary tumours (as examples, see Figure 3 and Figure 5). The uptake of [^99m^Tc]Tc-ADAPT6 in the primary tumours was significantly (*p* < 0.005, paired *t*-test) higher than the uptake of [^99m^Tc]Tc-(HE)_3_-G3 (Figure 5). Due to the high uptake of [^99m^Tc]Tc-ADAPT6 in a non-involved breast (Figure 4A), there was no significant difference in primary tumour-to-contralateral site ratios between tracers. All known nine lymph node metastases were also visualized with both tracers (see examples in Figure 7). There was no significant difference (*p* > 0.05, paired *t*-tests) between average uptake in primary tumours and lymph nodes metastases for the tracers, but an appreciable difference between uptakes was noticed for several lesions (Figure 8A,B). A large variation in metastatic uptake in the same patients has been observed for other tracers as well, such as [^68^Ga]Ga-ABY-025 affibody [29] and [^99m^Tc]Tc-NM-02 sdAb [24]. Quite likely, this variation reflects a well-known discordance between HER2 expression in primary breast cancer [5]. Overall, [^99m^Tc]Tc-ADAPT6 demonstrated a significantly higher uptake in extrahepatic soft tissue lesions (Figure 8). This might be an advantage for the visualization of small metastases because this alleviates the negative consequences of the partial volume effect. Thus, [^99m^Tc]Tc-ADAPT6 might be the preferable tracer for patient stratification for HER2-targeting therapy suitability. However, another potential area of HER2 imaging in clinics is the monitoring of a molecular response to HER2-targeting therapies, such as the downregulation of HER2 receptors. ADAPT6 and trastuzumab bind to the same epitope of HER2 [16], while the binding epitope of DARPin G3 is different [32]. A preclinical evaluation demonstrated that trastuzumab blocks the binding of [^99m^Tc]Tc-ADAPT6 to HER2-expressing cells in vitro but does not prevent the binding of [^99m^Tc]Tc-(HE)_3_-G3 [20]. Therefore, [^99m^Tc]Tc-(HE)_3_-G3 might be suitable for detecting the downregulation of HER2 in tumours in response to treatments, which include trastuzumab or its conjugates with drugs. The blocking of the epitope would not influence such imaging. This was evaluated in a pilot clinical study (NCT05412459). The tumour uptake of [^99m^Tc]Tc-(HE)_3_-G3 was measured before the neoadjuvant therapy of HER2–positive breast cancer using the TCbHP regimen (combination of docetaxel, carboplatin, trastuzumab and pertuzumab) and after 2 and 4 cycles of such therapy. The preliminary results suggest that the uptake of [^99m^Tc]Tc-(HE)_3_-G3 decreases in responsive tumours already after the second cycle of TCbHP. 

It is worth mentioning that both [^99m^Tc]Tc-ADAPT6 and [^99m^Tc]Tc-(HE)_3_-G3 were capable of visualizing hepatic metastases in two patients (Figure 9). In both cases, the uptake of [^99m^Tc]Tc-ADAPT6 and [^99m^Tc]Tc-(HE)_3_-G3 in liver metastases was higher than in the primary tumours (Figure 10), which can be explained by a higher blood flow or higher vasculature permeability in the liver. This elevated uptake in hepatic metastases enabled a clear visualization of metastases even with [^99m^Tc]Tc-(HE)_3_-G3, despite its higher uptake in the normal liver. However, it is unclear if this will be the case with more stringent statistics based on a larger cohort. Furthermore, both tracers visualized lung and numerous bone metastases in patient 1 (Figure 11). Although this finding is promising, no conclusions could be drawn from a single patient’s data. 

The preclinical development of scaffold protein-based targeting agents is rapidly expanding. Probes, which are based on DARPin, affibody and anticalin scaffolds, have been proposed for the radionuclide imaging of cancer-associated targets such as epithelial cell adhesion molecule (EpCAM) [33], check point protein B7-H3 [34], programmed death ligand 1 (PD-L1) [35], prostate-specific membrane antigen (PSMA) [36], insulin-like growth factor-1 receptor (IGF-1R) [37] and HER2 [38] during the last two years. Still, clinical data are available mainly for HER2-targeting affibody molecules [26,28,29,39,40,41]. Clinical validation has not been performed for the majority of the other scaffold-protein-based tracers, and the features of their biodistribution and targeting properties in humans have not been evaluated and compared. This complicates the selection of optimal scaffolds for the development of novel imaging probes. This study contributes to building clinical knowledge, which is essential in making informed decisions concerning the development of novel traces for molecular imaging.

## 5. Conclusions

Both [^99m^Tc]Tc-ADAPT6 and [^99m^Tc]Tc-(HE)_3_-G3 were capable of visualizing HER2-expressing breast cancer lesions, including primary tumours, lymph node metastases and, in two patients, hepatic metastases. A significantly higher uptake of [^99m^Tc]Tc-ADAPT6 in soft tissue lesions and its significantly lower uptake in the normal liver might be the advantages of this tracer in clinics. 

## Figures and Tables

**Table 1:** Patient Characteristics Before Injection with [^99m^Tc]Tc-ADAPT6 and [^99m^Tc]Tc-(HE)_3_-G3.

	Age (y)	HER2 Status in Primary Tumour (IHC ^a^)	Primary Tumour Status (ER/PgR/Ki67)	Primary Tumour Size (mm)	LN Size (mm)	Clinical Stage Before Imaging
1	61	3+ (ICH)	ER+; PgR+; Ki67 40%	20	24	IV (T4N3M1)
2	48	3+ (ICH)	ER+; PgR+; Ki67 18%	37	23	IIB (T2N1M0)
3	26	3+ (ICH)	ER+; PgR+; Ki67 45%	49	20	IIB (T2N1M0)
4	49	3+ (ICH)	ER+; PgR+; Ki67 20%	15	none	I (T1N0M0)
5	41	3+ (ICH)	ER+; PgR+; Ki67 45%	20	24	IV (T1N1M1)
6	65	3+ (ICH)	ER+; PgR+; Ki67 60%	21	37	IIB (T2N1M0)
7	59	3+ (ICH)	ER−; PgR−; Ki67 55%	13	16	IIA (T1N1M0)
8	55	3+ (ICH)	ER−; PgR−; Ki67 18%	28	30	IIB (T2N1M0)
9	38	3+ (ICH)	ER+; PgR+; Ki67 25%	38	12	IIB (T2N1M0)
10	65	3+ (ICH)	ER−; PgR−; Ki67 18%	17	none	I (T1N0M0)
11	63	3+ (ICH)	ER+; PgR+; Ki67 10%	20	none	I (T1N0M0)

^a^ IHC: immunohistochemistry.

## Data Availability

The data generated during the current study are available from the corresponding author upon reasonable request.
